# Staying and shifting patterns across IGT trials distinguish children with externalizing disorders from controls

**DOI:** 10.3389/fpsyg.2013.00899

**Published:** 2013-12-02

**Authors:** Isabela Sallum, Fernanda Mata, Débora M. Miranda, Leandro F. Malloy-Diniz

**Affiliations:** ^1^Laboratório de Investigações Neuropsicológicas, Instituto Nacional de Ciência e Tecnologia de Medicina Molecular, Faculdade de Medicina – Universidade Federal de Minas GeraisBelo Horizonte, Brazil; ^2^Faculty of Medicine, School of Psychology and Psychiatry, Monash UniversityMelbourne, VIC, Australia

**Keywords:** attention deficit hyperactivity disorder, oppositional defiant disorder, iowa gambling task, strategy, decision making, externalizing disorders

## Abstract

The Iowa Gambling Task (IGT) is the most widely instrument used in the assessment of affective decision-making in several populations with frontal impairment. The standard performance measure on the IGT is obtained by calculating the difference between the advantageous and the disadvantageous choices. This standard score does not allows the assessment of the use of different strategies to deal with contingencies of gain and losses across the task. This study aims to compare the standard score method used in IGT with a method that analyses the patterns of staying and shifting among different decks across the 100 choices, considering contingencies of choices with and without losses. We compared the IGT performance of 24 children with externalizing disorders (Attention Deficit Hyperactivity Disorder and Oppositional Defiant Disorder) and 24 healthy age-matched children. The analyses of the standard score across all blocks failed to show differences among children with externalizing disorders and control children. However, healthy children showed a pattern of shifting more from disadvantageous decks to advantageous decks and choosing more consecutive cards from the advantageous decks across all blocks, independently of the contingency of losses. On the other hand, children with externalizing disorders presented a pattern of shifting more from advantageous decks to disadvantageous ones in comparison to healthy children and repeatedly chose cards from the B deck across all blocks. This findings show that even though differences among groups might not be found when using the standard analyses, a different type of analysis might be able to show distinct strategies on the execution of the test.

## Introduction

Children diagnosed with Attention Deficit Hyperactivity Disorder (ADHD) have been characterized as poor decision makers whose response in decisions involving risk is guided by attractive immediate choices independent of their negative outcomes in the long term (Barkley, 1997). For instance, ADHD patients are at greater risk of accidental injuries in childhood (Byrne et al., 2003). In adolescence and adulthood, ADHD patients have been found to have an increased likelihood to impulsively quit a job (Halmøy et al., 2009), to express aggressive behaviors in response to driving related anger and crash-related outcomes (Richards et al., 2006), and experience antisocial activities and arrests as a consequence of illegal drug use (Barkley et al., 2004). Several studies have demonstrated the impulsive immediatist response style of ADHD children in a laboratory setting using the Iowa Gambling Task (IGT) and its child-friendly versions of this task (Garon and Moore, 2006; Malloy-Diniz et al., 2008; Masunami et al., 2009). Nevertheless, some studies did not find affective decision-making deficits in ADHD children using this same instrument (Geurts et al., 2006; Suhr et al., 2008; Hobson et al., 2011; Ibáñez et al., 2011).

The IGT (Bechara et al., 1994) is a well known worldwide measure of affective decision-making under uncertainty and it has become available as a clinical instrument in the past decade (Bechara, 2007). In the task, participants are given a $2000 loan of play money and are instructed to win as much money as possible by repeatedly choosing cards from four different decks. The expected value of the decks vary such that two decks are associated with high immediate gains, but repeated selections result in financial loss (disadvantageous decks, A and B). Conversely, the other two decks are associated with low immediate gains, but repeated selections result in financial gain in the long run (advantageous decks, C and D).

Standard measures frequently used for analyzing IGT performance combine the difference between total advantageous and disadvantageous cards selected throughout the task and the pattern of this difference according to five 20-block trials over the course of the 100 selections of cards (Bechara et al., 1998). Other outcome measures used for analysing IGT performance include total money won (van den Bos et al., 2006); total of cards selected on individual decks (Chiu and Lin, 2007); comparison between the number of cards selected from the decks A and C (low-frequency losses) and decks B and D (high-frequency losses) (Chiu and Lin, 2007); and analyses of deck selection in all the 100 trials vs. the last 50 trials (Rocha et al., 2011).

However, it has been demonstrated that the most used performance score (simple difference score between advantageous and disadvantageous choices) has important limitations (Buelow and Suhr, 2009; Ferguson et al., 2009; Visagan et al., 2012). These limitations are distinguished because it only takes into account long-term outcomes (Horstmann et al., 2012) and ignores the strategy used by the participant during the task. For instance, participants who do not adopt any strategy during the task might have a score close or even above zero if they choose cards randomly and do not show a preference for one of the decks. On the other hand, participants who choose predominantly cards from the disadvantageous decks in the first block of the IGT and demonstrate a slow and gradual preference for the advantageous decks over the task, can have a lower score compared to participants who choose randomly. Furthermore, it should be noted that all of these outcome measures mentioned above do not allow any interpretation of shifts between decks and stays. Even though a recent search for more detailed methods to analyze IGT performance in different clinical populations characterized by orbitofrontal cortex deficits has received attention in the past decade, to our knowledge no study has employed an analysis based on shift frequencies between the decks and stays to investigate IGT performance in ADHD children.

Our hypothesis about the inconsistent findings regarding the decision-making deficits in ADHD children might be at least partially explained by the often exclusive use of the standard net score to compare IGT performance between children with externalizing disorders and typical developing children. It should be noted that these conflicting findings raises questions about the appropriateness of this instrument in ADHD diagnosis (Buelow and Suhr, 2009) and should be investigated in a more detailed way. We hypothesized that ADHD children present difficulty in using the information about the gain and loss aspects of the decks to efficiently select cards from the advantageous decks throughout the task. As pointed by Meel et al. (2005), advantageous decision-making requires frequent monitoring and updating of current strategies to take into account new information. The examination of the appropriateness and success of performance plays an important role in determining and implementing behavioral adjustments (Ridderinkhof et al., 2004). Importantly, it has been suggested that online monitoring of external feedback may be relatively preserved in ADHD children (Meel et al., 2005; Groen et al., 2013), although they fail to properly utilize internal feedback to adjust their current response strategies.

Given these findings, it is important to compare the standard score method most used to analyze IGT performance with a method that analyzes the patterns of staying and shifting among different decks considering contingencies of choices with and without losses. This comparison could help to investigate whether this alternative analysis method is capable to characterize more accurately the decision-making deficits of children with externalizing disorders.

## Materials and methods

### Participants

Twenty-four children diagnosed with externalizing disorders from a public health service (Attention Deficit Hyperactivity Disorder and/or Oppositional Defiant Disorder; 6 girls; Mean age = 10.04 years, *SD* = 1.654) and 24 aged-matched controls (9 girls; Mean age = 10.29 years, *SD* = 1.546), all ranging from 7 to 14 years old, participated in the present study. Clinical diagnoses were done by a psychiatrist using the K-SADS-PL (Schedule for Affective Disorders and Schizophrenia for School-Age Children, Present and Lifetime Version; Kaufman et al., 1997). Of our clinical sample, 83% met criteria for ADHD only (20 children, 7 classified with Predominantly Inattentive subtype and 13 classified with Combined subtype), 4% met criteria for ODD only (1 boy) and 13% met criteria for both ADHD and ODD (3 children, 1 classified with Predominantly Hyperactive subtype and 2 classified with Combined subtype). The participants had similar socioeconomic backgrounds (as measured by the Brazilian Criterion of Economic Classification; see on the methods section), with predominantly middle to low socioeconomic status, and attended public schools, except for one boy in the clinical group who attended a private school. The children from the clinical group were restricted from their medication for 24 h before the assessment.

### Measures

#### The brazilian criterion of economic classification (CCEB)

Socioeconomic status was measured using the CCEB (Brazilian Research Enterprises Association; ABEP, 2008), a widely used measure of purchase power of families living in urban areas in Brazil. The questionnaire assesses available resources at home and the educational level of the householder, resulting in a scale ranging from 0 to 46. The families are further classified into eight economic classes, from top to bottom: A1 (42–46 points), A2 (35–41), B1 (29–34), B2 (23–28), C1 (18–22), C2 (14–17), D (8–16), and E (0–7). Our sample had a mean of 20.33 (*SD* = 5.164). Only one child from the control group was classified as being part of the class E. The others ranged from classes B2 to D. There were no differences amongst groups.

#### Standard IGT analyses

A computerized version of the IGT developed by Malloy-Diniz et al. (2007) for the Brazilian population was used. For the standard analyses of the IGT, the choices across the task are divided in 5 blocks with 20 trials each, and what is analysed is the proportion of choices in advantageous decks (C and D) minus disadvantageous decks (A and B) across the task.

#### Strategy use analyses—staying and shifting patterns in the IGT and deck preferences

In order to verify different strategies used by the two groups in the IGT, analyses of the patterns of staying and shifting among different decks across the 100 choices were done for each participant. For these analyses, we considered how many times each participant would choose to stay in a certain deck or shift to another deck according to the presence or absence of losses after each choice. Staying was defined as choosing the same deck immediately after this deck was chosen (for example, choosing the A deck right after this deck was chosen). Shifting was defined as choosing a different deck than the immediate previous one (for example, choosing the B deck after choosing the A deck). Different levels of complexity were encompassed, since considering only the number of overall choices of staying and shifting, to considering patterns of staying and shifting according to different types of decks (advantageous × disadvantageous; high-frequency losses decks × low-frequency losses decks), different decks (A,B,C,D) and contingencies of losses (with × without losses). The number of overall choices in each separate deck was also analysed in order to test if the groups would differ in their preferences.

Further analyses were run in order to identify the different strategies used by the groups across the 5 different blocks. For that, eight conditions of shifting/staying were considered based on the division of cards amongst advantageous and disadvantageous cards: (1) staying in an advantageous deck without losses; (2) staying in an advantageous deck after losses; (3) staying in a disadvantageous deck without losses; (4) staying in a disadvantageous deck after losses; (5) shifting from an advantageous deck without; (6) shifting from an advantageous deck after losses; (7) shifting from a disadvantageous deck without losses; (8) shifting from a disadvantageous deck after losses.

During the task, the choices each participant makes defines whether this person is more prone to receive a punishment or not (for example, choosing predominantly decks A and C will lead to a higher chance of losing conditions, while choosing predominantly decks B and D will lead to smaller chances of losing condition), therefore the analyses were done using proportions. For example, for condition 1 it was considered the raw number of choices for staying in an advantageous deck without losses, divided by the overall number of choices without losses. This was done for all of the conditions. Such method of analysis also allows the comparison between conditions without losses and after losses.

## Results

To analyse the standard measure of the IGT (number of advantageous choices minus number of disadvantageous choices at each block), a 2 (groups) × 5 (blocks) mixed model analyses of variance (ANOVA) was run. Huynh Feldt corrections were used since the sphericity assumptions were violated. No main effects or interactions were found to be significant. The effects of group fell short from being significant, *F*_(1, 46)_ = 0.052, *p* = 0.821. These results thus, show that, according to this analysis, children with externalizing disorders presented a similar performance when compared to healthy controls.

Analyses of overall differences in shifting/staying and deck preferences among the groups were run using Kolmogorov-Smirnov test, showing 16 statistically different variables (see Table 1). These first analyses showed that healthy children overall chose more to stay in any given deck in comparison to children with externalizing disorders. Healthy children would also stay more in advantageous decks, regardless of the contingency of presence or absence of losses, and would shift more from disadvantageous decks to advantageous ones whenever there were penalties (losses). On the other hand, the clinical group was more prone to shift from advantageous decks to disadvantageous ones and chose more from the deck B in comparison to healthy controls. When analyzing high-frequency losses decks (Hfl) and low-frequency losses decks (Lfl), again the control group would stay more in any of these conditions in comparison to the clinical group. This is probably an effect of the overall preference of the control group in staying in any condition in comparison to the clinical group. Healthy children would also shift more from a Lfl to another Lfl, showing a preference over the clinical group for choosing cards with low frequency of losses.

**Table 1:** **Analyses of all variables that differed significantly amongst the groups**.

**Variable**	**Healthy controls**			***ADHD/ODD***			***K–S***	***p***
	***Mdn***	***M***	***SD***	***Mdn***	***M***	***SD***		
Deck preference								
B deck	25.5	26.63	6.006	29	29.88	6.622	**2.14**	**0.032**
Overall staying and shifting								
Staying in any loss condition	22.5	23.38	5.02	20	21.83	17.135	1.443	0.031
Staying after losses	6	5.79	2	3.5	4.88	4.848	1.443	0.031
Staying and shifting—Adv. and Disadv.								
Staying in Adv. without losses	8.5	9.08	3.844	4.5	7.96	8.518	1.588	0.013
Staying in Adv. after losses	3	2.88	1.484	1	1.75	2.575	1.732	0.005
Shifting from Disadv. after losses to Adv.	8	8.04	2.274	5	5.33	2.914	1.732	0.005
Shifting from Disadv. after losses to Disadv.	3	3.67	1.971	6	5.88	2.643	**1.443**	**0.031**
Staying and shifting—Hfl and Lfl								
Staying in Hfl	10	9.29	3.085	5	8.25	8.543	1.443	0.031
Staying in Hfl after losses	4.5	4.25	1.726	2	3.33	3.985	1.443	0.031
Staying in Lfl	14	14.08	4.652	12.5	13.88	11.372	1.443	0.031
Staying in Lfl without losses	12	34.54	4.16	10.5	33.29	10.115	1.443	0.031
Shifting from Lfl without losses to Lfl	4	11.92	1.393	3.5	8.71	1.472	1.732	0.005
Staying and shifting for each deck								
Staying in C after losses	2	2.13	1.361	0	1.21	2.245	1.876	0.002
Shifting from A after losses to D	3.5	3.71	1.899	1	2.04	1.601	1.443	0.031
Shifting from A after losses to B	2.5	3	1.865	5	5.08	2.125	**1.588**	**0.013**
Shifting from D without losses to B	5	5.88	2.997	2.5	4.17	2.973	**1.588**	**0.013**

Mdn, median; m, mean; sd, standard deviation; K–S, Kolmogorov–Smirnov; p, p-value; Adv, advantageous decks (C and D); Disadv, disadvantageous decks (A and B); Hfl, high-frequency losses decks (A and C); Lfl, low-frequency losses decks (B and D). The bold values indicate the choices that were more frequent for the clinical group in comparison to the control group.

ADHD, Attention Deficit Hyperactivity Disorder; ODD, Oppositional Defiant Disorder.

**Figure 1:**
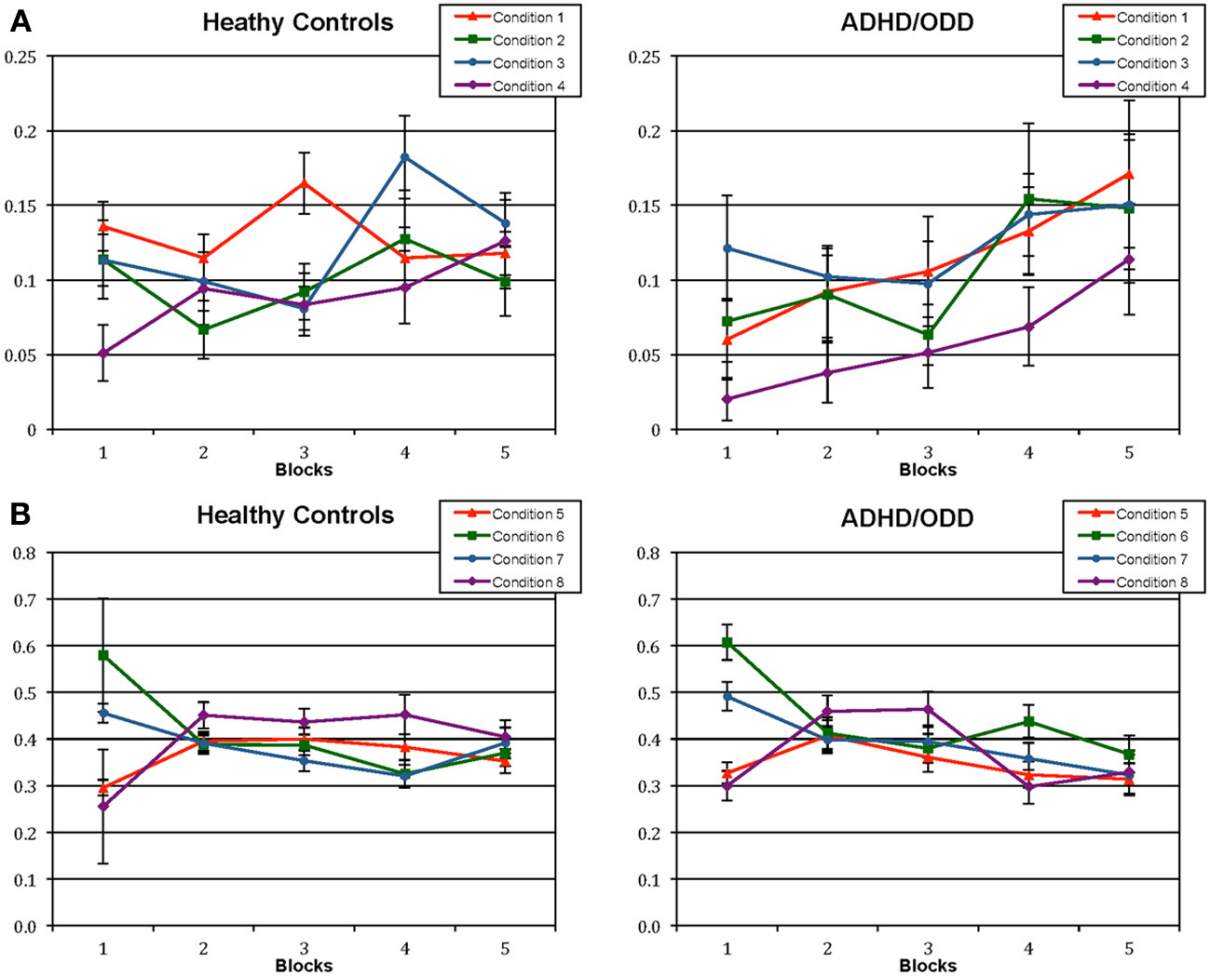
**Proportion of choices in each of the eight conditions of staying and shifting in advantageous and disadvantageous decks for healthy controls and children with externalizing disorders (ADHD and ODD). (A)** shows the choices in the staying conditions; Condition (1) staying in an advantageous deck without losses/ overall choices without losses; Condition (2) staying in an advantageous deck after losses/ overall choices after losses; Condition (3) staying in a disadvantageous deck without losses/ overall choices without losses; Condition (4) staying in a disadvantageous deck after losses/ overall choices after losses. **(B)** shows the choices in the shifting conditions: Condition (5) shifting from an advantageous deck without losses/ overall choices without losses; Condition (6) shifting from an advantageous deck after losses/ overall choices after losses; Condition (7) shifting from a disadvantageous deck without losses/ overall choices without losses; Condition (8) shifting from a disadvantageous deck after losses/ overall choices after losses. *ADHD, Attention Deficit Hyperactivity Disorder; ODD, Oppositional Defiant Disorder*.

A 2 (groups) × 4 (conditions 1–4) × 5 (blocks) three-way mixed models analyses of variance (ANOVA) was conducted to analyse differences in staying across blocks. Sphericity was not assumed and Huynh-Feldt corrections were used. There was a main effect of conditions, *F*_(2.313, 106.390)_ = 4.277, *p* = 0.012, and blocks *F*_(3728, 171.478)_ = 7.557, *p* < 0.001, and only a borderline significant two-way interaction between groups × blocks, *F*_(3728, 171.478)_ = 1.936, *p* = 0.111. Further analyses were run to compare the choices amongst groups for each condition separately using a Bonferroni-corrected *p*-value of 0.0125 (one analysis for each condition) in order to control for type 1 error. There was only a borderline significant interaction between groups × blocks for condition 1 (staying in an advantageous deck without losses), *F*_(4, 184)_ = 2.629, *p* = 0.036, showing that the pattern of choices for this condition would only change across blocks for the clinical group. As can be seen in Figure 1, children from the clinical group would increase their choices for staying in an advantageous without losses across the blocks, whilst children from the control group would already start the task choosing this condition more often. Furthermore, analyses of simple effects were run verifying each condition for each group separately (Bonferroni-corrected *p*-value of 0.0125). For some of the analyses, sphericity was not assumed and Huynh-Feldt corrections were used. For the control group, the number of choices for staying in a disadvantageous deck without losses increased across blocks, *F*_(3,059, 70.361)_ = 4.045, *p* = 0.01, which was not observed for the clinical group since they already would start the task choosing this condition more often. On the other hand, the clinical group presented a borderline significant increase across blocks in choosing to stay in a disadvantageous deck after losses, *F*_(4, 92)_ = 2.838, *p* = 0.029, while this was not observed for the control group. Further analyses comparing each condition for each group were run, but no significant differences were found.

To analyse the shifting conditions, a 2 (groups) × 4 (conditions 5 to 8) × 5 (blocks) three-way mixed models analyses of variance (ANOVA) was conducted. Sphericity was not assumed and Huynh-Feldt corrections were used. There was a main effect of conditions, *F*_(2.681, 123.329)_ = 7.235, *p* < 0.001, and blocks *F*_(3.939, 181.2010)_ = 9.622, *p* < 0.001, two two-way interaction between blocks and groups, *F*_(3.939, 181.2010)_ = 3.020, *p* = 0.020, and blocks and conditions, *F*_(9.341, 429.677)_ = 11.656, *p* < 0.001, and a higher order interaction between blocks, conditions and groups, *F*_(9.341, 429.677)_ = 1.898, *p* = 0.048. Since the main and two-ways interactions are contained in the higher order interaction, analyses of simple effects were run focusing on this interaction. Analyses of simple effects were run to compare each condition amongst each other using a Bonferroni-corrected *p-value* for 6 comparisons (*p* = 0.008), but there was no significant interaction between condition × groups for any of the analyses. Furthermore, analyses were run to compare the choices amongst groups for each condition separately (Bonferroni-corrected *p-value* of 0.0125). There was a significant blocks × groups interaction for choices of shifting from a disadvantageous deck after losses (Condition 8), *F*_(4, 184)_ = 3.509, *p* = 0.009, showing that the pattern of choices for this condition would have greater changes across blocks for the clinical group, presenting a decrease. When analyzing each condition separately for each group (four analyses, Bonferroni-corrected *p-value* of 0.0125), it was shown that both groups would decrease their choices of shifting from an advantageous deck across the task [control group: *F*_(4, 92)_ = 16.308, *p* < 0.001; clinical group: *F*_(4, 92)_ = 8.887, *p* < 0.001]; both would also present a decrease in choosing to shift from a disadvantageous deck without losses [control group: *F*_(4, 92)_ = 4.649, *p* < 0.001; clinical group: *F*_(4, 92)_ = 5.205, *p* = 0.002], and both presented a change across blocks in choosing to shift from a disadvantageous deck without losses [control group: *F*_(4, 92)_ = 6.492, *p* < 0.001; clinical group: *F*_(4, 92)_ = 7.728, *p* < 0.001], however, the pattern of choices for this condition was different for each group, once the control group increased their choices in this condition in the beginning of the task and then maintained the a constant number of shifts, while the clinical group presented an increase of choices in the beginning of the task, followed by a decrease in the ending, as shown in Figure 1.

When both the staying and shifting analyses are taken together, it's possible to see that the children with externalizing disorders start the task shifting more and they take longer to establish a pattern of staying in a deck, even thought in the beginning they choose to stay more in disadvantageous decks without losses. The clinical group also shows a significant decrease in shifting across the blocks. On the other hand, even though the control group also shows a decrease in shifts across blocks, they already start the task staying more in advantageous conditions and their pattern of shifts do not change as much as for the clinical group. Overall, the clinical group seemed to present more changes in the pattern of shifting and staying across blocks than the control group, and seem to start using a strategy of shifting less and staying more in a deck in the last blocks.

**Figure 2:**
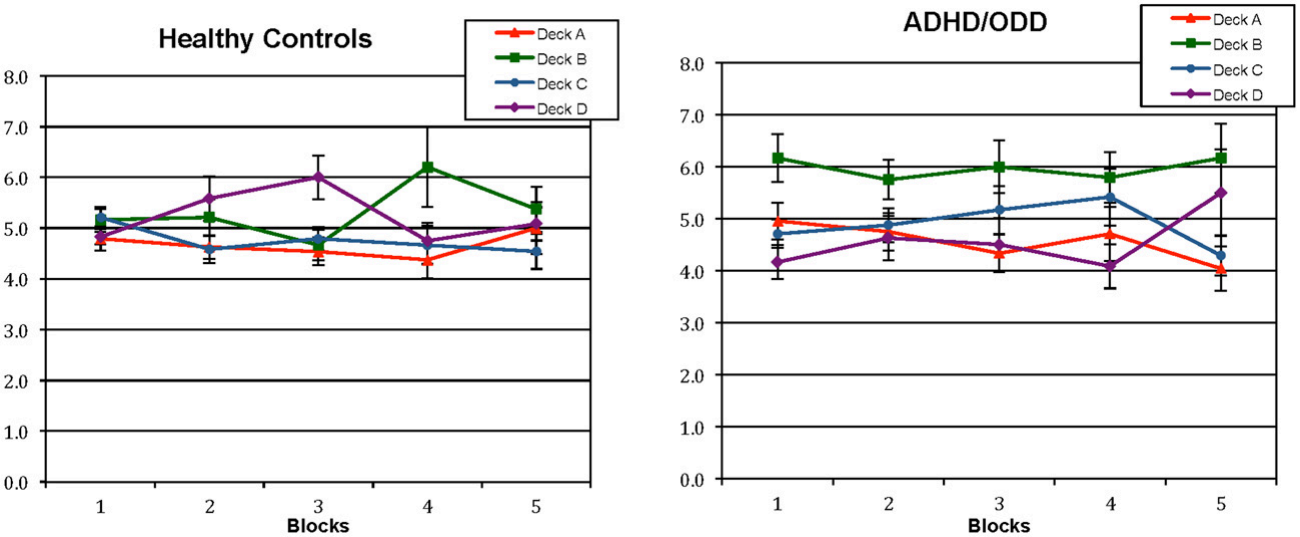
**Average sum of selections from individual decks across each block during the performance of the Iowa Gambling Task for healthy controls and children with externalizing disorders (ADHD and ODD).** *ADHD, Attention Deficit Hyperactivity Disorder; ODD, Oppositional Defiant Disorder*.

Since the overall analyses of shifting, staying and deck preferences showed that the clinical group presented a preference for the deck B in comparison to controls, a 2 (groups) × 4 (decks; A,B,C,D) × 5 (blocks) Three-Way mixed models ANOVA was conducted to analyse preference for a specific deck across the task. Greenhouse-Geisser corrections were used since the sphericity assumptions were violated. No main effects or interactions were found to be significant. To further analyze a possible effect over the different groups, a 4(decks) × 5 (blocks) Two-Way mixed models ANOVA was conducted separately for each group. Sphericity was assumed. For the control group, there was no significant main effects or interactions. However, for the clinical group, there was a main effect of decks, *F*_(3, 69)_ = 4.883, *p* = 0.004. Analyses of simple effects Bonferroni-corrected for 6 comparisons (*p* = 0.008) showed that there was a preference for choices in deck B over deck A, *F*_(1, 23)_ = 14.796, *p* = 0.001, and B over D, *F*_(1, 23)_ = 8.822, *p* = 0.007. The average number of choices for each deck across the blocks is shown in Figure 2, for the clinical and control groups.

## Discussion

The present study aimed to analyse strategy use in the performance of the IGT amongst healthy children and children with externalizing disorders, comparing the standard score analyses used in the IGT with a more detailed analyses based on shift-frequencies between the decks and staying-frequencies in each deck. In the present study, standard performance analysis did not reveal any statistically significant difference between children with externalizing disorders and healthy controls. This finding suggests that the clinical group may not be impaired in affective decision-making, which is not in agreement with the real-life decision-making problems observed in children with externalizing disorders. Nevertheless, differences in the strategies adopted by the participants of the different groups on the execution of the task could be observed when the analysis based on shift-frequencies between the decks and staying-frequencies in each deck were used.

Analyzing shifts and staying frequencies amongst decks showed overall that children from the control group shifted from disadvantageous decks to advantageous ones more frequently than the clinical group, while children from the clinical group shifted more from the disadvantageous decks whenever they had losses to another disadvantageous one. Overall, staying in either types of deck was noted statistically more often in typical children compared to children with externalizing disorders across the task. These findings indicate that healthy controls might choose more cards from the advantageous decks than the clinical group, even though it could not be observed in the standard performance analysis. When analyzing the overall choices of shifting and staying, the clinical group did not seem to present a clear strategy, which can be evidenced by the fact that shifts from the deck D without losses to the deck B were statistically more frequent in participants from the clinical group compared to controls. However, when observing the performance throughout the task, the clinical group seemed to start choosing to shift less and stay more in a deck in the last blocks. This shows that they might have established a strategy in the ending of the task, even though it is not necessarily a good one, since they start staying more in both advantageous and disadvantageous decks and start shifting less from disadvantageous decks. It is important to notice that the control group presented a significant change in the shifting conditions across blocks, but their pattern of staying and shifting did not change as much throughout the task when compared to the clinical group. These results do not show a clear strategy emerging throughout the task, instead they show that the control group already establish a pattern of choices in the beginning of the task. This corroborates with the overall analyses showing that in the entire task, healthy children choose to stay more in any given deck when compared to the clinical group.

As we hypothesized, children and adolescents with externalizing disorders seem to have some difficulty to use information about the gain/loss aspects from past choices to advantageously select cards throughout the task. The shifting patterns of the clinical group, as observed on the overall analyses, showed that they choose more than controls to shift to a disadvantageous deck. This could also possibly be explained by a difficulty in discriminating between the “good” and the “bad” decks of the task, as proposed by Meel et al. (2005). In a study investigating decision-making and autonomic response to reinforcement in ADHD children, Meel et al. (2005) demonstrated that this clinical population presented difficulty in discriminating between positive and negative outcomes associated with affective evaluation.

In addition to analyzing the shift frequencies between decks and stays, more detailed methods of analysis to investigate IGT performance have also focused on preferences for individual decks during the task. By employing such an analysis in the present study, it was found that healthy controls did not present a clear preference for a specific deck, whereas children with externalizing disorders demonstrated a preference for the deck B throughout the task.

Toplak et al. (2005) showed that both ADHD and healthy controls demonstrated a preference for cards from the deck B to cards from other decks, which is partially similar to our findings. Moreover, Horstmann et al. (2012) showed that healthy young adults were more prone to choose cards from the decks B and D in the IGT, rather than cards from the decks A and C, because the first ones present a lower frequency of losses. Overall, for the decks A and C, 50% of all the choices present a loss, while for the decks B and D, only 10% of the choices present a loss, although those losses are higher. The authors argued that the frequency of punishment, rather than the magnitude of it, seems to control the gambling behavior on the IGT.

In the present study, it was shown that healthy children would choose to stay in any of these types of decks more often than the clinical group, probably reflecting their overall tendency to stay in any given deck more often than children with externalizing disorders. Furthermore, the preference manifested by the clinical group for the deck B, but not for the deck D, can probably be explained by the magnitude of reinforcement, since the deck B presents a reinforcement that is the double of the deck D, even though it's punishment is 10 times higher than that of deck D. Either a working memory issue, or a higher sensitivity of children with externalizing disorders to reinforcement than to punishment might explain such preference pattern.

In consideration of the first possible explanation for these findings, it should be noted that considering that deck B demands less tracking of expected values since losses are less frequent, selection of cards from this deck may reflect less recruitment of working memory as highlighted by Toplak et al. (2005). It has been shown that ADHD children and adolescents perform worse than controls in tasks evaluating working memory (Nikolas and Nigg, 2013), and so do children with ODD only and comorbid ADHD and ODD (Rhods et al., 2011). Importantly, cognitive research has shown that working memory plays an important role in subserving the active mental representation of an individual's self-regulatory goals and related ways by which these goals can be achieved (Miller and Cohen, 2001).

In consideration of the second explanation for these findings, it has been shown that ADHD children seem to be oversensitive to rewards and to be less sensitive to punishments (Luman et al., 2005). Luman et al. (2008) investigated the performance of ADHD children in a decision-making task involving choosing an advantageous deck vs. disadvantageous decks in two conditions: one in which the frequency of penalties increased and another, in which the magnitude of penalties increased. The authors found that ADHD children performed similar to controls in the condition of increasing frequency of penalties, but did worse whenever the magnitude of penalties increased. This indicates that ADHD children are sensitive to frequency, but not to the magnitude of losses. The preference for the deck B found in this analyses, in conjunction to the analyses of staying and shifting shows that although the children from the clinical group stop to consistently stay in a specific deck after the second block they maintain the deck B as a reference and choose to shift from other cards to this card.

Compared to controls, children with externalizing disorders also chose cards from the deck B statistically more frequently. Toplak et al. (2005) found that ADHD adolescents selected more cards from the deck B and fewer cards from the deck D compared to controls, which partially confirms our findings. The wide age range of our group can possibly explain the absence of preference for a specific deck demonstrated by the healthy control group in the present study. Possibly, a preference for the deck B would be encountered if only children and adolescents over 10 years old were investigated. In fact, in a study aiming to measure affective decision-making in typical children and adolescents 8- to 17- years old, Smith et al. (2012) found that younger children failed to show a preference for either deck whereas IGT performance of children from ages 10 to 13 was characterized by persistent selections of cards from the disadvantageous decks.

This study has important limitations. Unfortunately, intelligence was not assessed in the current study, although it is unlikely that intelligence may have affected the present findings since it has been shown that it is not related to affective decision-making (Mata et al., 2013). The wide age range of the present study is also a limitation, since IGT performance in children and adolescents is known to be influenced by development (Smith et al., 2012). Another limitation is that the number of participants is small and the clinical group is very heterogeneous. It's possible that children with different ADHD subtypes, ODD only, or comorbid ADHD with ODD, would show different strategies in the execution of the IGT. For example, Toplak et al. (2005) showed that participants with ADHD of the Combined Subtype chose the decks B and D more frequently than children with ADHD of the Inattentive subtype, while the latter chose more the decks A and C comparison. Regardless of these limitations, this study showed that even though differences in affective decision-making between children with externalizing disorders and controls were not found using IGT standard performance analyses, considering how the task was executed and the strategies used, a more detailed analyses might be more precise in identifying patterns of performance across this task. Different authors suggested other types of analyses of the IGT, such as the analysis of dyadic moves across blocks as proposed by Ferguson et al. (2009). This considers the number of times participants chose one type of deck choice (advantageous or disadvantageous) followed by other advantageous or disadvantageous choice. This type of analysis also encompasses strategy-use during the performance of the IGT. The combination of different types of analyses of the IGT with other cognitive measures, such as working memory tasks, might further help clarifying performance on this task.

### Conflict of interest statement

The authors declare that the research was conducted in the absence of any commercial or financial relationships that could be construed as a potential conflict of interest.
